# Supplementary material for: Membrane recruitment of the polarity protein Scribble by the cell adhesion receptor TMIGD1
Source: Commun Biol. 2023 Jul 10;6:702. doi: 10.1038/s42003-023-05088-3 (PMC10333293; doi:10.1038/s42003-023-05088-3)
Supplement: Supplementary file 2 — Supplementary Information FINAL [file 42003_2023_5088_MOESM2_ESM.pdf]

Supplementary Information

Membrane recruitment of the polarity protein Scribble by the cell adhesion receptor TMIGD1

Eva-Maria Thüring[1,†], Christian Hartmann[1,†], Janesha C. Maddumage[2,†], Airah Javorsky[2], Birgitta E. Michels[1], Volker Gerke[3], Lawrence Banks[4], Patrick O. Humbert[2], Marc Kvansakul[2,*], Klaus Ebnet[1,5,*]

[1]Institute-associated Research Group "Cell adhesion and cell polarity", Institute of Medical Biochemistry, ZMBE, University of Münster, Münster, Germany;
[2]Department of Biochemistry & Chemistry, La Trobe Institute for Molecular Science, La Trobe University, Melbourne, Vic, Australia;
[3]Institute of Medical Biochemistry, ZMBE, University of Münster, Münster, Germany;
[4]International Centre for Genetic Engineering and Biotechnology, Padriciano 99, I-34149 Trieste, Italy
[5]Cells-in-Motion Interfaculty Center, University of Münster, 48419 Münster, Germany;

[†]These authors contributed equally to this work

[*]Authors for correspondence
Klaus Ebnet, e-mail: ebnetk@uni-muenster.de; ORCID: 0000-0002-0417-7888
Marc Kvansakul, e-mail: m.kvansakul@latrobe.edu.au; ORCID: 0000-0003-2639-2498

Supplementary Figures

Supplementary Figure 1: 2Fo-Fc electron density maps of Scrib PDZ1 in complex with TMIGD1 PBM peptide

Supplementary Figure 2: Localization of endogenous Scrib in TMIGD1-transfected HEK293 cells

Supplementary Figure 3: Membrane recruitment of Scrib PDZ domain mutants by TMIGD1

Supplementary Figure 4: Co-Localization of TMIGD1 with Scrib/PDZ-CAAX at Golgi membranes

Supplementary Figure 5: Localization of endogenous Scribble in ΔD1-MDCKII and recruitment of Scrib/PDZ, Scrib/LRR by ΔD1-TMIGD1 in MDCK cells

Supplementary Figure 6: Unprocessed scans of blots and gels shown in Figs 1b – d; 2b – e; 4b

Supplementary Tables

Supplementary Table 1: Data collection and refinement statistics

Supplementary Table 2: Isothermal titration calorimetry binding parameters

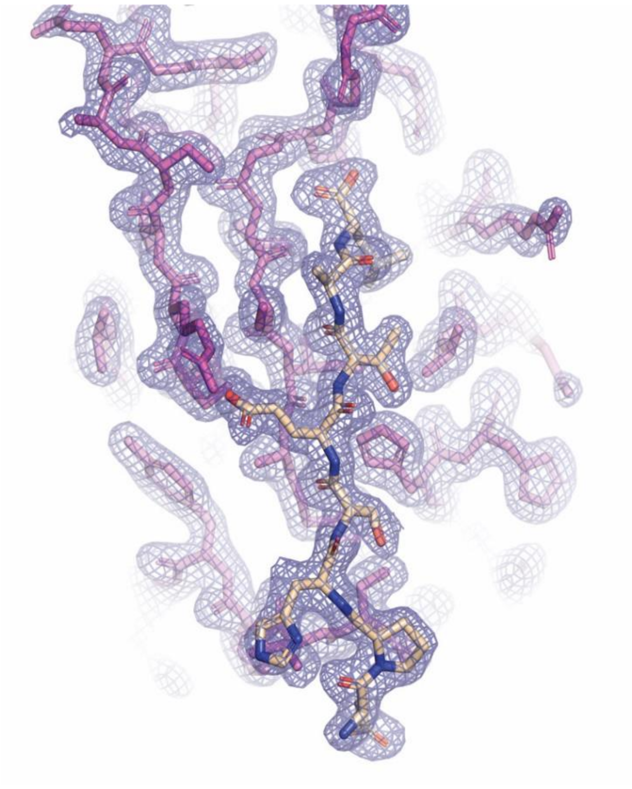

Supplementary Figure 1: 2Fo-Fc electron density maps of Scribble PDZ1 in complex with TMIGD1 PBM peptide. Electron density map is shown as blue mesh and contoured at 1.5 σ, encompassing the binding groove of Scribble PDZ domain 1 (light magenta) in complex with TMIGD1 PBM peptide (cream) shown as sticks.

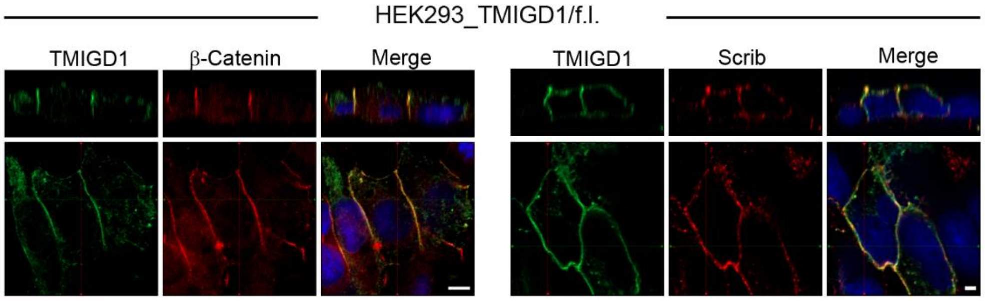

Supplementary Figure 2: Lateral localization of TMIGD1 in HEK293 cells. HEK293 cells transfected with TMIGD1 were stained with antibodies against TMIGD1 and β-catenin (left panels) or TMIGD1 and Scrib (right panels) as indicated. Cells were analyzed by confocal microscopy. The top panels depict XZ projections. Scale bars: 5 µm (left panels), 2 µm (right panels).

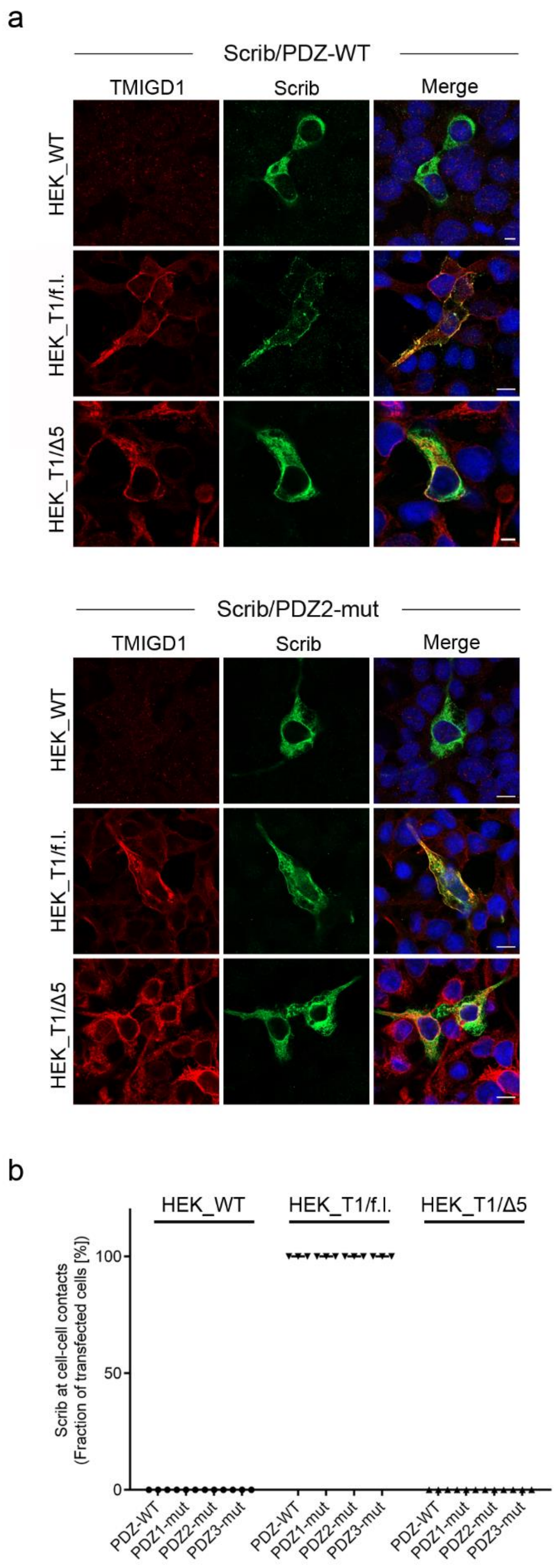

Supplementary Figure 3: Recruitment of Scrib-PDZ mutants by TMIGD1. (**a**) HEK293 cells, either untransfected (HEK-WT) or stably transfected with TMIGD1/full length

(HEK_T1/f.l.) or with TMIGD1 lacking the PBM (HEK_T1/Δ5) were transiently transfected with Scrib PDZ domain 1 to 4 constructs, either wildtype PDZ domains (Scrib/PDZ-WT) or PDZ domains 1, 2 or 3 mutated (Scrib/PDZ1-mut, Scrib/PDZ2-mut, Scrib/PDZ3-mut, depicted in Fig. 4A). Cells were stained for TMIGD1 and for the indicated Scrib PDZ mutant constructs (anti-His tag). (**b**) Quantification of Scrib recruitment to cell-cell contacts. The dot plot graph shows the fraction of cells with Scrib localization at cell-cell contacts. Scrib/PDZ-WT: n = 60 (HEK_WT), n = 61 (HEK_T1/f.l.), n = 60 (HEK_T1/Δ5); Scrib/PDZ1-mut: n = 60 (HEK_WT), n = 61 (HEK_T1/f.l.), n = 60 (HEK_T1/Δ5); Scrib/PDZ2-mut: n = 62 (HEK_WT), n = 61 (HEK_T1/f.l.), n = 60 (HEK_T1/Δ5); Scrib/PDZ3-mut: n = 61 (HEK_WT), n = 62 (HEK_T1/f.l.), n = 61 (HEK_T1/Δ5); N = 3 independent experiments, represented by individual dots. Scale bars: 5 µm.

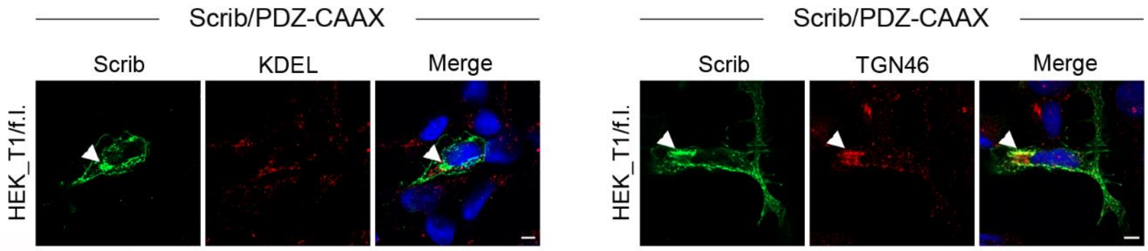

Supplementary Figure 4: Scrib/PDZ-CAAX is localized at the Golgi apparatus. HEK293 cells stably transfected with TMIGD1/full length (HEK_T1/f.l.) and transiently transfected with the Scrib/PDZ_CAAX construct were stained for the Scrib/PDZ-CAAX construct (anti-Myc tag) and for markers for the endoplasmic reticulum (KDEL) or for the Golgi apparatus (TGN46). Scale bars: 5 µm.

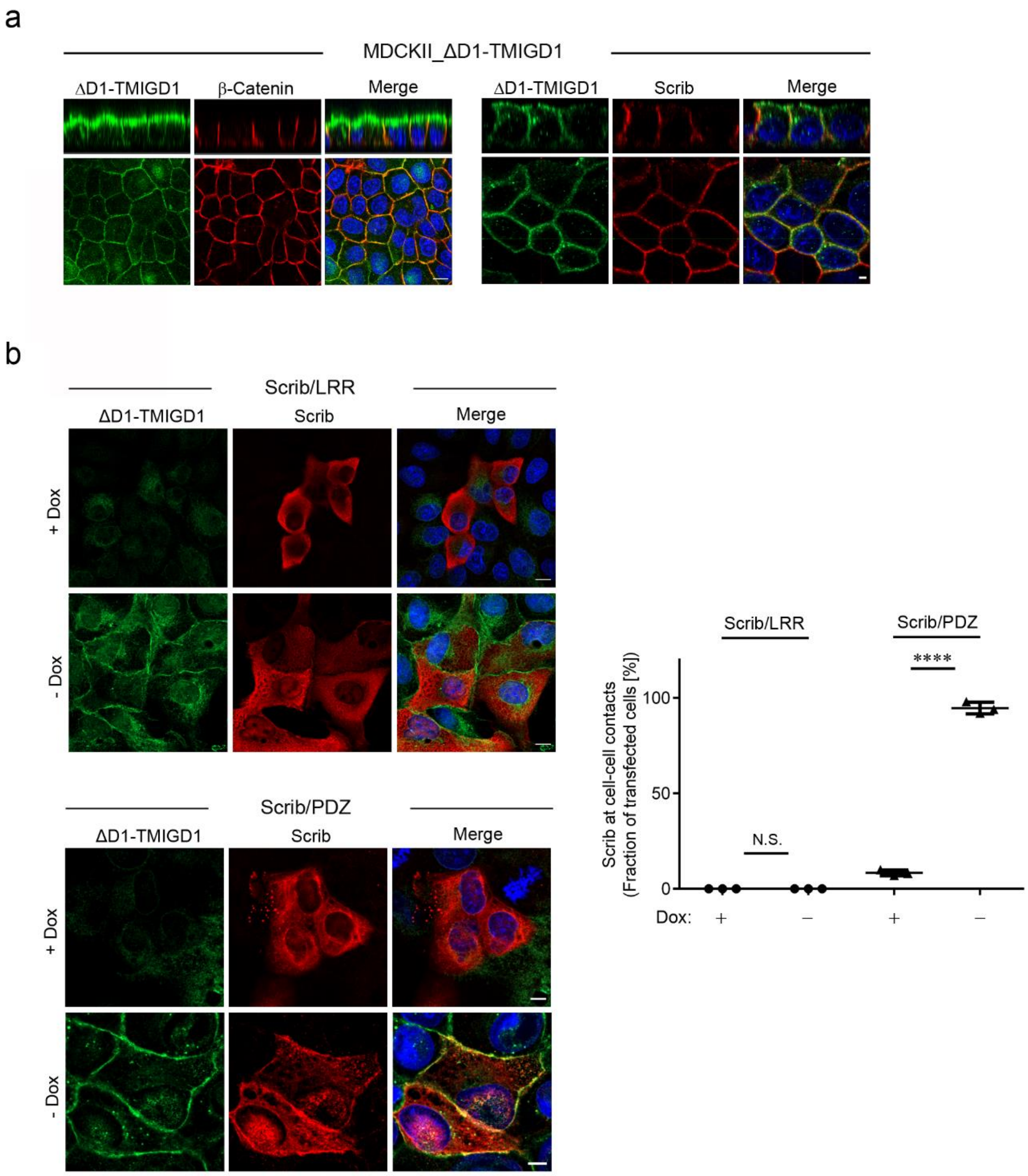

Supplementary Figure 5: TMIGD1 recruits Scrib in polarized epithelial cells. (**a**) Localization of endogenous Scrib in MDCKII cells stably expressing Flag-tagged ΔD1-TMIGD1 under a doxycycline (Dox)-regulated promoter. Cells were stained with antibodies against TMIGD1 (anti-Flag) and β-catenin (left panels) or TMIGD1 (anti-Flag) and Scrib (right panels) and analyzed by confocal microscopy. The top panels depict XZ projections. Scale bars: 10 µm (left panel), 2 µm (right panel). Note that ΔD1-TMIGD1 is localized both at the apical membrane and at lateral cell-cell junctions where it partially co-localizes with β-catenin and Scrib. (**b**) Localization of Scrib/LRR and Scrib/PDZ in ΔD1-TMIGD1-expressing MDCKII cells. Cells were transiently transfected with the indicated Scrib mutant constructs and were either

left uninduced (+ Dox) or were induced to express ΔD1-TMIGD1 (- Dox). Cells were stained for TMIGD1 (anti-Flag) and Scrib (anti-Myc). Scale bars: 10 µm (Scrib/LRR), 5 µm (Scrib/PDZ). The dot plot shows the fraction of cells with Scrib localization at cell-cell contacts. For each condition 150 Scrib-transfected cells were analyzed, data is derived from N = 3 independent experiments (represented by individual symbols). Statistical analysis was performed with unpaired Student's t-test. Data are presented as mean values ±SD. N.S., not significant, ****, $p < 0.0001$.

Supplementary Table 1: Data collection and refinement statistics.

	HS_Scrib_PDZ1: HS_TMIGD1
Data collection	
Space group	I 4_1 22
Cell dimensions	
a, ***b***, ***c*** (Å)	53.664 53.664 215.703
α, β, γ (°)	90.00 90.00 90.00
Wavelength (Å)	0.9537
Resolution (Å)*	43.01-1.9 (1.968-1.9)
R_{sym} or R_{merge}*	0.07706 (0.7481)
I / σI*	29.91 (4.17)
CC(1/2)*	1 (0.94)
Completeness (%)*	99.97 (99.92)
Multiplicity*	26.2 (27.1)
Refinement	
Resolution (Å)*	43.01-1.9 (1.968-1.9)
No. reflections*	12998 (1247)
R_{work} / R_{free}	0.1961/0.2223
No. non-hydrogen atoms	871
Protein	809
Ligand/ion	-
Water	62
B-factors	
Protein	32.60
Ligand/ion	-
Water	41.37
R.m.s. deviations	
Bond lengths (Å)	0.011
Bond angles (°)	1.28
Ramachandran plot (%)	
Favored	100.00
Allowed	0.00
Disallowed	0.00

* Values in parentheses refer to the highest resolution shell.

Supplementary Table 2: Isothermal titration calorimetry binding parameters. Each of the values was calculated from at least three independent experiments.

Scrib/PDZ vs TMIGD1 peptide	K_D (µM)	N	ΔH (cal.mol^{-1})	TΔS (cal.mol^{-1}K^{-1})
Scrib_ PDZ1	18.17 ± 1.80	0.95 ± 0.07	2869.25 ± 228.1	19.923 ± 1.1
Scrib_ PDZ2	NB	-	-	-
Scrib_ PDZ3	9.12 ± 0.67	0.93 ± 0.04	13480 ± 351.6	-22.13 ± 1.1
Scrib_ PDZ4	NB	-	-	-

Supplementary Figure 6:
Unprocessed scans of blots and gels shown in Figs 1b – d; 2b – e; 4b

Fig.1B

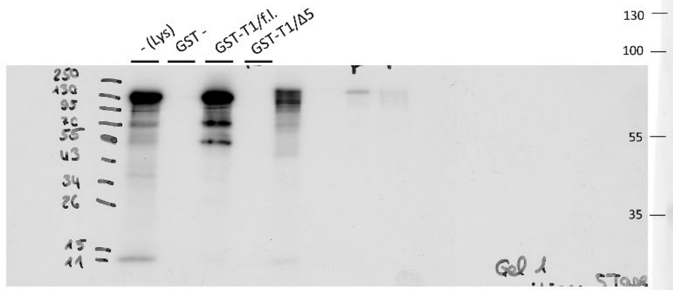

Antibody: Scrib

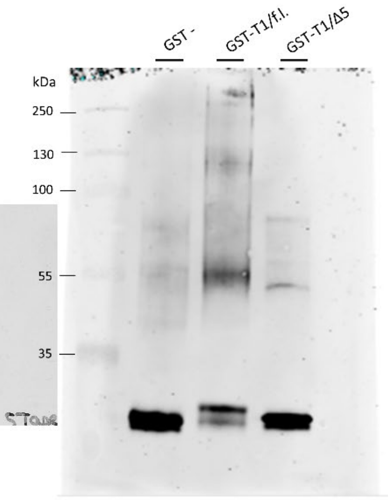

Antibody: GST

Fig.1C, left

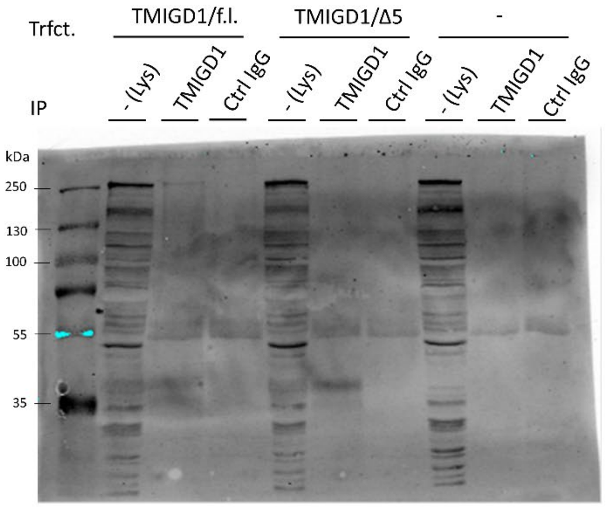

Antibody: TMIGD1

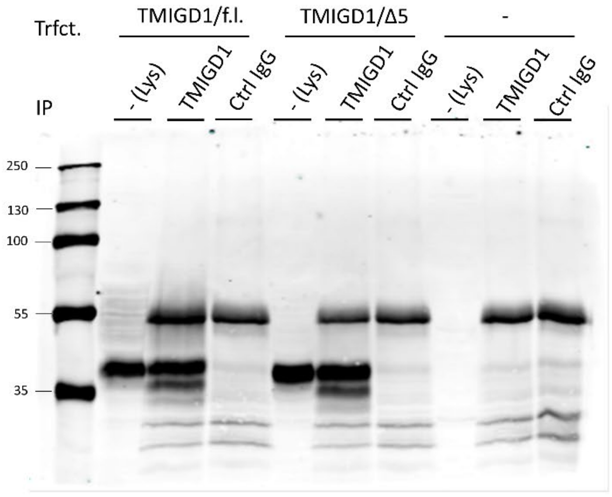

Antibody: Scrib

Fig.1C, right

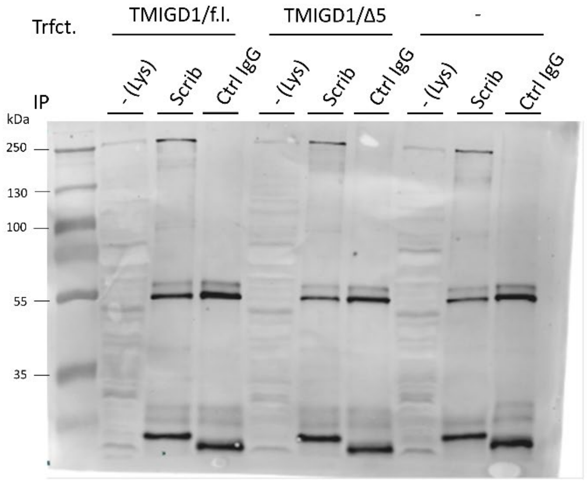

Antibody: Scrib

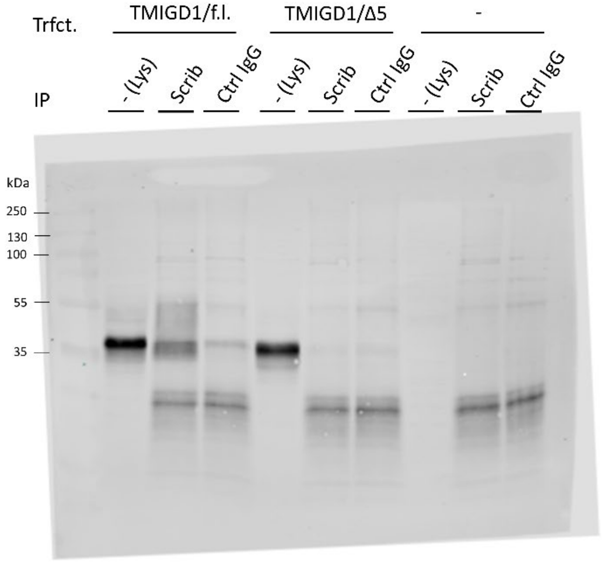

Antibody: TMIGD1

Fig.1D

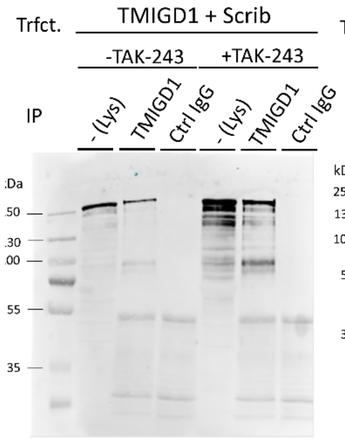

Antibody: Scrib

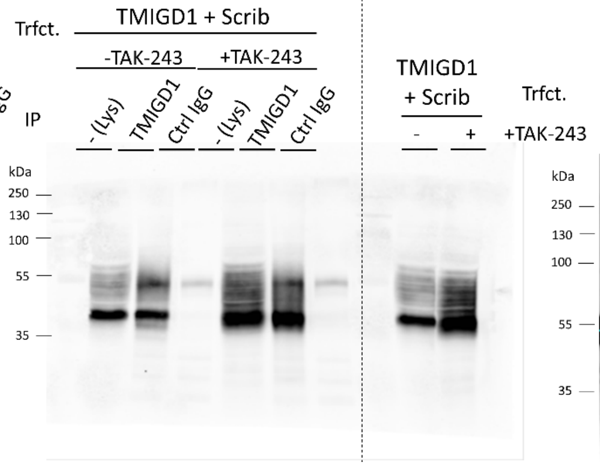

Antibody: TMIGD1

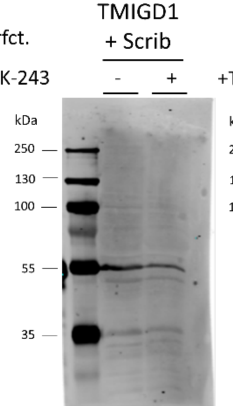

Antibody: GAPDH

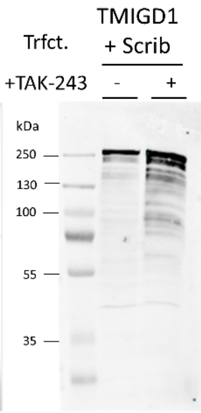

Antibody: Scrib

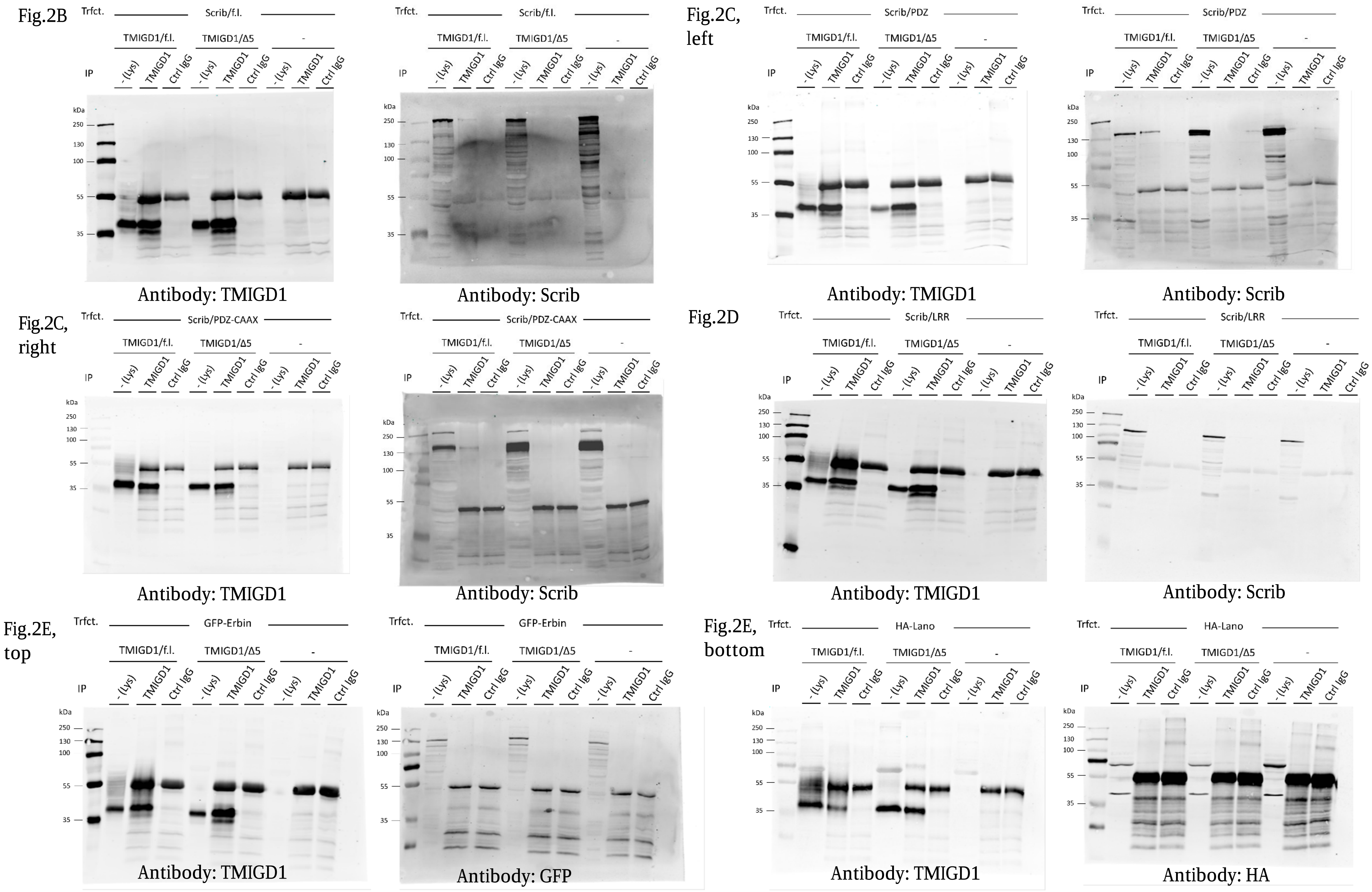

Fig.2B
Trfct.
Scrib/f.l.
TMIGD1/f.l.
TMIGD1/Δ5
-
IP
- (Lys)
TMIGD1
Ctrl IgG
kDa
250
130
100
55
35
Antibody: TMIGD1
Antibody: Scrib
Fig.2C, left
Scrib/PDZ
Antibody: TMIGD1
Antibody: Scrib
Fig.2C, right
Scrib/PDZ-CAAX
Antibody: TMIGD1
Antibody: Scrib
Fig.2D
Scrib/LRR
Antibody: TMIGD1
Antibody: Scrib
Fig.2E, top
GFP-Erbin
Antibody: TMIGD1
Antibody: GFP
Fig.2E, bottom
HA-Lano
Antibody: TMIGD1
Antibody: HA

Fig.4B

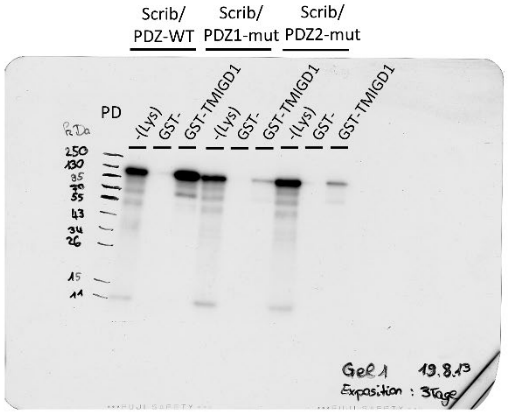

Antibody: Scrib

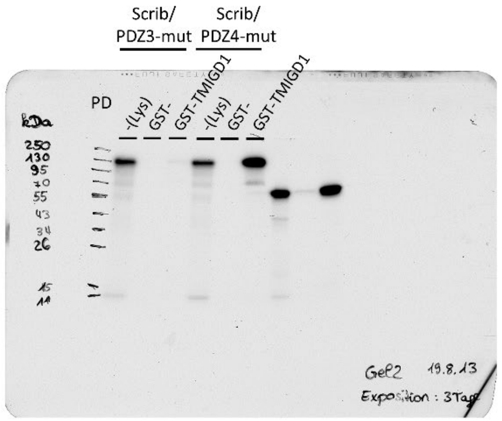

Antibody: Scrib

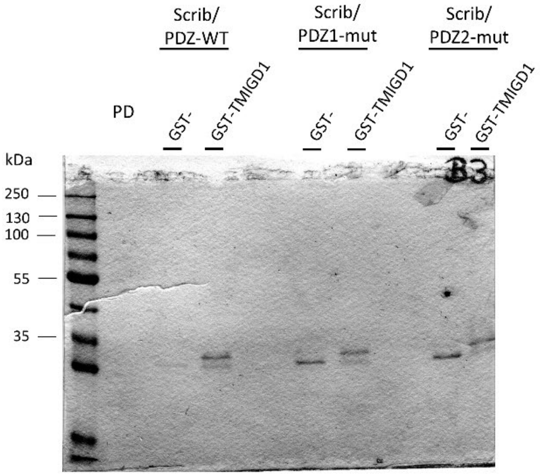

Antibody: GST

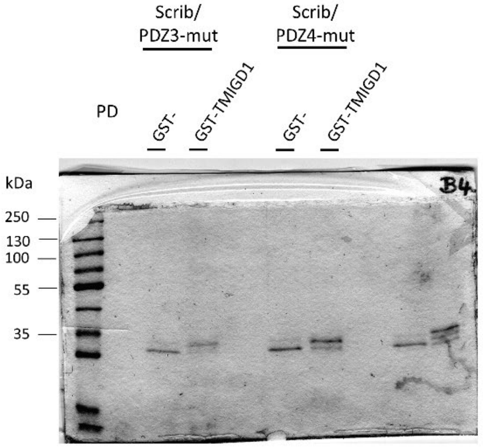

Antibody: GST
