# Supplementary material for: Membrane recruitment of the polarity protein Scribble by the cell adhesion receptor TMIGD1
Source: Commun Biol. 2023 Jul 10;6:702. doi: 10.1038/s42003-023-05088-3 (PMC10333293; doi:10.1038/s42003-023-05088-3)
Supplement: Supplementary file 3 — Description of Additional Supplementary Files [file 42003_2023_5088_MOESM3_ESM.pdf]

Description of Additional Supplementary Files

File name: Supplementary Data 1

Description: Numerical source data behind all graphs in the manuscript.
