# Supplementary material for: Membrane recruitment of the polarity protein Scribble by the cell adhesion receptor TMIGD1
Source: Commun Biol. 2023 Jul 10;6:702. doi: 10.1038/s42003-023-05088-3 (PMC10333293; doi:10.1038/s42003-023-05088-3)
Supplement: Supplementary file 5 — Reporting Summary [file 42003_2023_5088_MOESM5_ESM.pdf]

nature portfolio

Corresponding author(s): Marc Kvansakul, Klaus Ebnet

Last updated by author(s): Jun 27, 2023

Reporting Summary

Nature Portfolio wishes to improve the reproducibility of the work that we publish. This form provides structure for consistency and transparency in reporting. For further information on Nature Portfolio policies, see our Editorial Policies and the Editorial Policy Checklist.

Statistics

For all statistical analyses, confirm that the following items are present in the figure legend, table legend, main text, or Methods section.

n/a	Confirmed	
☐	☒	The exact sample size (*n*) for each experimental group/condition, given as a discrete number and unit of measurement
☐	☒	A statement on whether measurements were taken from distinct samples or whether the same sample was measured repeatedly
☐	☒	The statistical test(s) used AND whether they are one- or two-sided<br>*Only common tests should be described solely by name; describe more complex techniques in the Methods section.*
☒	☐	A description of all covariates tested
☐	☒	A description of any assumptions or corrections, such as tests of normality and adjustment for multiple comparisons
☐	☒	A full description of the statistical parameters including central tendency (e.g. means) or other basic estimates (e.g. regression coefficient) AND variation (e.g. standard deviation) or associated estimates of uncertainty (e.g. confidence intervals)
☒	☐	For null hypothesis testing, the test statistic (e.g. *F*, *t*, *r*) with confidence intervals, effect sizes, degrees of freedom and *P* value noted<br>*Give P values as exact values whenever suitable.*
☒	☐	For Bayesian analysis, information on the choice of priors and Markov chain Monte Carlo settings
☒	☐	For hierarchical and complex designs, identification of the appropriate level for tests and full reporting of outcomes
☒	☐	Estimates of effect sizes (e.g. Cohen's *d*, Pearson's *r*), indicating how they were calculated

Our web collection on statistics for biologists contains articles on many of the points above.

Software and code

Policy information about availability of computer code

Data collection	- Odyssey Infrared Imaging System Application Software Version 3.0<br>- ZEN 2.1 (blue edition)<br>- AIMLESS 8.0.004<br>- xia2/DIALS 3.12.2
Data analysis	- Odyssey Infrared Imaging System Application Software Version 3.0<br>- ZEN 2.3 lite (blue edition)<br>- Microsoft Excel 2016<br>- ImageJ<br>- Imaris (Bitplane, Version 9.1.2)<br>- GraphPad Prism 6.0 Software, San Diego, CA<br>- Phenix 1.20.1-4487<br>- PyMol 2.5.4_420

For manuscripts utilizing custom algorithms or software that are central to the research but not yet described in published literature, software must be made available to editors and reviewers. We strongly encourage code deposition in a community repository (e.g. GitHub). See the Nature Portfolio guidelines for submitting code & software for further information.

Data

Policy information about availability of data

All manuscripts must include a data availability statement. This statement should provide the following information, where applicable:

- Accession codes, unique identifiers, or web links for publicly available datasets
- A description of any restrictions on data availability
- For clinical datasets or third party data, please ensure that the statement adheres to our policy

All data generated or analyzed during this study are included in this published article and its supplementary information files, or are available from the corresponding author upon reasonable request.

Human research participants

Policy information about studies involving human research participants and Sex and Gender in Research.

Reporting on sex and gender	NA
Population characteristics	NA
Recruitment	NA
Ethics oversight	NA

Note that full information on the approval of the study protocol must also be provided in the manuscript.

Field-specific reporting

Please select the one below that is the best fit for your research. If you are not sure, read the appropriate sections before making your selection.

☒ Life sciences ☐ Behavioural & social sciences ☐ Ecological, evolutionary & environmental sciences

For a reference copy of the document with all sections, see nature.com/documents/nr-reporting-summary-flat.pdf

Life sciences study design

All studies must disclose on these points even when the disclosure is negative.

Sample size	Statistical analyses for pre-determination of sample sizes were not performed. Sample sizes were estimated on the basis of similar assay systems reported in the literature. Details regarding sample sizes and number of replicates are provided in the Methods section and figure legends.
Data exclusions	No data were excluded from the analysis.
Replication	Experiments were performed at least three times. All attempts of replication were successful.
Randomization	NA (randomization was not necessary in this study).
Blinding	NA (blinding was not necessary in this study).

Reporting for specific materials, systems and methods

We require information from authors about some types of materials, experimental systems and methods used in many studies. Here, indicate whether each material, system or method listed is relevant to your study. If you are not sure if a list item applies to your research, read the appropriate section before selecting a response.

Materials & experimental systems

n/a	Involved in the study	
☐	☒	Antibodies
☐	☒	Eukaryotic cell lines
☒	☐	Palaeontology and archaeology
☒	☐	Animals and other organisms
☒	☐	Clinical data
☒	☐	Dual use research of concern

Methods

n/a	Involved in the study	
☒	☐	ChIP-seq
☒	☐	Flow cytometry
☒	☐	MRI-based neuroimaging

Antibodies

Antibodies used

All antibodies used in this study are described in detail in the Methods section. The information includes supplier name, catalogue number, clone name and lot number (if applicable). In addition, the dilutions at which the antibodies were used in specific applications is provided.
Mouse mAb anti-α-Tubulin (Sigma-Aldrich, clone B-5-1-2, #T5168, IF 1:500, WB 1:10.000);
Goat pAb anti-Myc (SantaCruz #sc-789G, IF 1:500, WB 1:500);
Mouse mAb anti-Myc 9E10 (Evan et al (1985) Mol. Cell. Biol. 5: 3610; IF 1:500, WB 1:500);
Mouse mAb anti-GFP (Takara, #632375, WB 1:500);
Rabbit pAb anti-Hemagglutinin (HA) (Sigma-Aldrich, #H6908, WB: 1:500);
Rabbit pAb anti-Flag (Sigma-Aldrich, #F7425, IF 1:500);
Mouse mAb anti-6xHis (proteintech #66005-1-Ig, IF 1:500);
Rabbit pAb anti-TMIGD1 (Sigma-Aldrich, #HPA021946, IF 1:500);
Rabbit pAb anti-TGN46 (abcam #ab50595, IF 1:500);
Rabbit pAb anti-KDEL (ThermoFisher Scientific #PA1-013, IF 1:2.500);
Mouse mAb anti-β-catenin (BD-TransductionLabs #610153, IF 1:500);
Mouse mAb anti-Scribble (SantaCruz #sc-55543, IF 1:500);
Rabbit pAb anti-TMIGD1 Affi1662/1663 (Hartmann et al (2022) Sci. Signal. 15: eabm2449; IF 1:500, WB 1:500)
IRDye 800CW Donkey anti-Rabbit IgG (LI-COR Biosciences #926-32213, WB 1:10.000);
IRDye 680CW Donkey anti-mouse IgG (LI-COR Biosciences #926-68072, WB 1:10.000);
Donkey anti-Mouse IgG (H+L) Alexa Fluor 594 (ThermoFisher Scientific #A-21203, IF 1:800);
Donkey anti-Rabbit IgG (H+L) Alexa Fluor 594 (ThermoFisher Scientific #A-21207, IF 1:800);
Donkey anti-Rabbit IgG(H+L) Alexa Fluor 488 (ThermoFisher Scientific #A-21206, IF 1:800);
Donkey anti-Mouse IgG (H+L) Alexa Fluor 488 (Dianova/Jackson ImmunoResearch Europe Ltd #715-545-150, IF 1:800);
Donkey anti-Mouse IgG (H+L) Alexa Fluor 647 (Dianova/Jackson ImmunoResearch Europe Ltd #715-605-150, IF 1:800);

Validation

Commercially available primary antibodies directed against TMIGD1 have been validated by IF analysis using RNA interference. Validations of other commercially available antibodies can be found at the suppliers websites as detailed below.
1. Mouse mAb anti-α-Tubulin (Sigma-Aldrich, clone B-5-1-2, #T5168): https://www.sigmaaldrich.com/DE/de/product/sigma/t5168;
2. Goat pAb anti-Myc (SantaCruz #sc-789G): validated for specificity in WB and IF analysis (Hartmann et al (2022) Sci. Signal. 15: eabm2449);
3. Mouse mAb anti-Myc 9E10 (Evan et al (1985) Mol. Cell. Biol. 5: 3610): validated for specificity in WB and IF analysis (https://www.scbt.com/de/p/c-myc-antibody-9e10);
4. Mouse mAb anti-GFP (Takara, #632375): https://www.takarabio.com/products/antibodies-and-elisa/fluorescent-protein-antibodies/green-fluorescent-protein-antibodies;
5. Rabbit pAb anti-Hemagglutinin (HA) (Sigma-Aldrich, #H6908): https://www.sigmaaldrich.com/DE/de/product/sigma/h6908;
6. Rabbit pAb anti-Flag (Sigma-Aldrich, #F7425): https://www.sigmaaldrich.com/DE/de/product/sigma/f7425;
7. Rabbit pAb anti-TMIGD1 (Sigma-Aldrich, #HPA021946): https://www.sigmaaldrich.com/DE/de/product/sigma/hpa021946;
8. Rabbit pAb anti-TMIGD1 Affi1662/1663: validated for specificity in WB and IF analysis by ectopic expression of TMIGD1 in TMIGD1-deficient HEK293 cells (Hartmann et al (2022) Sci. Signal. 15: eabm2449)

Eukaryotic cell lines

Policy information about cell lines and Sex and Gender in Research

Cell line source(s)	The source of each cell line is stated in the manuscript.
Authentication	Cell lines used were not authenticated. Cell lines used in the study are not listed in the ICLAC register.
Mycoplasma contamination	Cell lines were tested for mycoplasma contamination on a monthly basis. All cell lines used in this study were negative for mycoplasma contamination.
Commonly misidentified lines (See ICLAC register)	Commonly misidentified cell lines were not used in this study.
